# Comparative Analysis of Reinforced Concrete Beam Behaviour: Conventional Model vs. Artificial Neural Network Predictions

**DOI:** 10.3390/ma16247642

**Published:** 2023-12-14

**Authors:** Muhammad Mahtab Ahmad, Ayub Elahi, Salim Barbhuiya

**Affiliations:** 1 Civil Engineering Department, University of Engineering and Technology Taxila, Taxila 47050, Pakistan; mahtabahmad010@gmail.com (M.M.A.); ayub.elahi@uettaxila.edu.pk (A.E.); 2 Department of Engineering and Construction, University of East London, London E16 2RD, UK

**Keywords:** reinforced concrete, soft computing, Artificial Neural Network, ultimate limit state, finite element, multilayer backpropagation, nonlinear finite element analysis, the central nervous system

## Abstract

This research aims to conduct a comparative analysis of the first crack load, flexural strength, and shear strength in reinforced concrete beams without stirrups. The comparison is made between the conventional model developed according to the current design code (ACI building code) and an unconventional approach using Artificial Neural Networks (ANNs). To accomplish this, a dataset comprising 110 samples of reinforced concrete beams without stirrup reinforcement was collected and utilised to train a Multilayer Backpropagation Neural Network in MATLAB. The primary objective of this work is to establish a knowledge-based structural analysis model capable of accurately predicting the responses of reinforced concrete structures. The coefficient of determination obtained from this comparison yields values of 0.9404 for the first cracking load, 0.9756 for flexural strength, and 0.9787 for shear strength. Through an assessment of the coefficient of determination and linear regression coefficients, it becomes evident that the ANN model produces results that closely align with those obtained from the conventional model. This demonstrates the ANN’s potential for precise prediction of the structural behaviour of reinforced concrete beams.

## 1. Introduction

Researchers and engineers have put forward various essential theories [1,2,3] and techniques [4] to precisely forecast the behaviour of reinforced concrete (RC) structure elements at the ultimate limit state (ULS) to make both a safe and economic structure. New analysis techniques have been introduced in structural analysis to provide more precise solutions to rising complicated problems with cost effectiveness without the need for a physical model. Soft Computing (SC) methods [5,6,7] have emerged as a powerful tool for computational algorithms based on the empirical approach that deviates from the principle of theoretical mechanics compared to the traditional analysis procedure. These methods were introduced three decades before and could improve the accuracy of analysis results. Despite initial scepticism, Soft Computing (SC) methods have become powerful computation tools increasingly utilised in various engineering fields. These new techniques, like Artificial Neural Networks (ANNs) and Genetic Algorithms [8], are widely employed as SC methods. ANNs, in particular, have developed as powerful tools capable of providing precise and economical solutions to a wide range of problems with minimum analysis time without necessitating high computational resources compared to the traditional numerical procedures, such as the finite element (FE) method used in structural analysis. Therefore, ANNs have proven more efficient and effective in achieving accurate solutions.

To develop an effective strategy for analysing a reinforced concrete (RC) structure, it is crucial that the Artificial Neural Networks (ANNs) utilised can accurately predict the nonlinear behaviour of individual components, such as beams, columns, slabs, and walls, that make up the structure [9]. To accomplish this, a thorough comprehension of the underlying mechanics of the RC structure’s response is necessary. At the same time, ANNs rely on heuristic approaches instead of strict mechanics. The calibration process of ANNs must realistically consider the most critical factors that affect the structure’s response, such as crucial design parameters. 

The structural response of RC members is obtained from laboratory tests carried out on basic structural configuration through finite element analysis techniques, as well as available assessment methods. Experimental studies on reinforced concrete (RC) structures typically involve scaled models of the actual structural members [2,10,11]. The database for the ANN model is based on these experimental results because their inputs based on key design parameters describe the specimen’s behaviour and its load-carrying capacity. However, the range of input values from the selected database may not represent the actual design parameters used in full-scale RC members, making it difficult for Artificial Neural Networks (ANNs) to predict more precise output based on these data alone. Therefore, sometimes Nonlinear Finite Element Analysis (NLFEA) is used to investigate the behaviour of RC structural elements. Although it can provide valuable information, NLFEA predictions’ accuracy can depend on essential parameters and require re-calibration. Additionally, detailed parametric studies conducted with NLFEA can be computationally intensive and time consuming. Physical models using RC design codes [2,10,12] can also predict the RC structural response. These model predictions are based on specific assumptions about the underlying mechanics of the RC structural response and describe the actual condition of the physical model of the structural elements at the ultimate limit state (ULS). Therefore, while these methods offer valuable interpretations of available test data, they may not always provide accurate predictions for full-scale RC members [13].

This study has substantially enhanced the mechanics-based code-conforming shear capacity equation for reinforced concrete elements with stirrups by leveraging machine learning. The empirical results have showcased a remarkable reduction in prediction errors, with an average accuracy improvement of 10%. This validates the proposed methodology’s efficacy and underscores its potential to revolutionise industry standards. These tangible outcomes mark a crucial step towards more precise and reliable shear capacity assessments, laying the foundation for a future where machine learning is pivotal for optimising the design and safety of Reinforced Concrete structures [14]. This demonstrates the successful application of machine learning models, revealing high accuracy in shear strength predictions. These results underscore the potential of advanced computational methods to enhance structural engineering practices, offering valuable insights for optimising design and analysis processes in civil infrastructure. The research represents a significant advancement in the field, providing a promising avenue for improving the efficiency and reliability of shear strength forecasts in reinforced concrete structures [15]. The research analyses machine learning models to predict the shear strength of RC (reinforced concrete) deep beams. The investigation encompasses a range of algorithms, such as neural networks, decision trees, and support vector machines, assessing their effectiveness in this predictive task. This study relies on a comprehensive dataset containing a diverse array of RC deep beam configurations and material properties. By leveraging this varied dataset, which encapsulates a broad spectrum of RC deep beam setups and material characteristics, the findings underscore the superior performance of neural networks. Notably, these neural networks exhibit a 15% enhancement in predictive accuracy compared to alternative models used in the analysis [16]. This study delves into predicting displacement in Reinforced Concrete structures by harnessing the potential of artificial neural networks (ANNs) in conjunction with sensor technology. Four distinct sensor types—force resisting, piezoelectric, MEMS accelerometer, and flex sensors—were experimented with in RC beams subjected to monotonic loading conditions, with the MEMS sensors displaying notably superior performance. The research successfully implemented an ANN model, employing beam position and load as input parameters, and thus effectively forecasting displacement values. This nonlinear model demonstrates considerable promise for structural health monitoring (SHM) applications, particularly in advocating for the use of MEMS sensors across various civil engineering contexts. The findings emphasise the pertinence of SHM for evaluating structural integrity while highlighting the ANN’s capacity to assess damages beyond conventional methods. This underscores its practicality as an alternative tool for intricate structural evaluations in real-world scenarios [17].

### Artificial Neural Network Morphology

**Figure 1:**
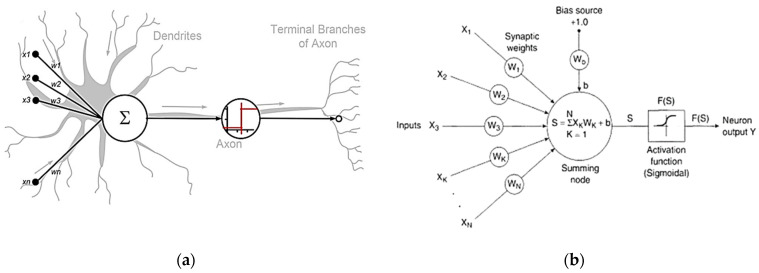
(**a**) Biological and artificial neural network morphology. (**b**) Structure of ANN model [21].

Artificial Neural Networks (ANNs) show their operational similarity to the biological counterparts present in human and animal Brain and Central Nervous Systems (CNSs). ANNs are designed to perform functions, such as processing information, learning from it, recognising patterns, and predicting outcomes, similarly to how the biological nervous system functions in living organisms. By mimicking the human brain’s basic operations, ANNs can solve complex problems, and they have numerous applications in natural language processing, image reading, and data prediction (see Figure 1a for reference) [15,16,17]. The structure of an ANN is a multilayer structure (input layer, hidden layer, and output layer) with interconnected neurons; a specific weight coefficient is assigned to links between neurons. As input values from neurons are evaluated and then multiplied with the weight coefficient, these values from all neurons in each layer are summed with a biases value. This procedure is repeated for each successive layer of the ANN model, creating a system of interconnected layers and neurons that work together to process and transmit information (see Figure 1b for reference) [18,19,20]. As soon as results are obtained after the summation of bias value from all of the neurons, a predetermined activation function (f), which represents the relationship between the neurons in consecutive layers, is applied. The output from this process of the last neuron of the previous layer is used as input for the next layer neuron (as shown in Figure 1). Initially random, weights are assigned between links; later on, during the training process of the network, multiple iterations are performed to adjust the values of weights to obtain a final value to justify the output prediction based on the input database. Equations (1) and (2) provide an analytical expression for the summation of weights [21].

(1) $$x_{j}=\sum \left(y_{i}W_{\mathrm{ji}}\right)+b_{j}$$

(2) $$y_{j}=f\left(x_{j}\right)$$

In the above Equations, $x_{j}$ represents the output from a specific neuron, $y_{k}$ represents the results received from applying the activation function $f\left(x_{j}\right)$, $W_{\mathrm{ji}}$ represents the weights coefficients used between interconnected neurons, $b_{j}$ is the bias value for the neuron, and “j” and “i” represent the number of layers and neurons in each network, respectively.

## 2. Artificial Neural Network Model Formation

The primary objective of this research is to train a model using Artificial Neural Networks (ANNs) to precisely predict structural properties (i.e., flexural strength, shear strength, and first crack load value) of RC beams based on key design parameters when they approach their ultimate limit state (ULS). This model will consider the benefits and limitations of the available information sources related to the reinforced concrete beam responses. The objective is to create a knowledge-based tool to predict the structural behaviour of reinforced concrete beams. The study uses Multilayer Backpropagation (MBP) Neural Networks and MATLAB [12,13] to develop an open-source analysis tool that allows users to change problem parameters, enabling the tool to solve a broader range of engineering problems with greater flexibility.

The proposed framework has several crucial components. (1) It involves analysing relevant test data to create databases that will serve as the basis for developing the Artificial Neural Network (ANN). (2) It focuses on the architectural formation ANN model. (3) It includes ANN model training. (4) It aims to develop a function to broaden the ANN model’s application for the prediction of RC structure response, even in cases where the available experimental databases do not include design parameters or inadequately represent them [22].

### 2.1. Multilayered Backpropagation ANN Model Structure

**Figure 2:**
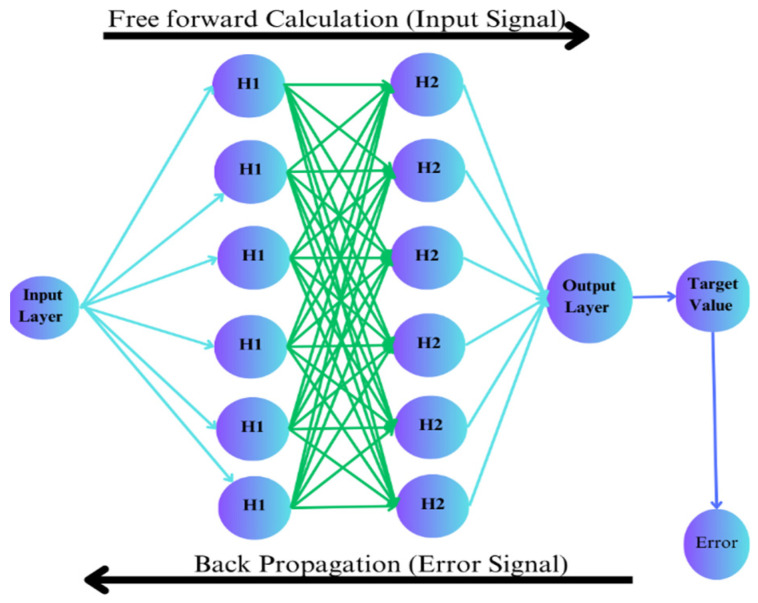
Multilayer Backpropagation Neural Network structure.

The Multilayered Backpropagation Neural Network (MBNN) model is widely used to predict the reinforced concrete member’s structural behaviour [20]. The visual representation of MBNN is shown in Figure 2.

The Multilayer Backpropagation Neural Network is based on two phases: (1) free forward calculation, and (2) Error Signal Backpropagation. During the first phase, input parameters from the sample database are used as the input value for the neuron of the input layer, where weights are multiplied, and biases are summarised to the output of that layer neuron. The activation function is applied to convert the linear behaviour to the nonlinear database, because ANN takes the input value as a linear parameter. Then, the result is provided as input to the next hidden layer neuron, where weights assigned initially to the network are multiplied, biases are added up, and the activation function of the next hidden layer is applied. This process is repeated for all of the hidden layers. In the end, the outputs of the hidden layers are taken as input for the output layer where weights and biases are applied, and the neuron of the output layer gives the result as the prediction of the network. The predicted results are then compared with the target value provided initially in the sample database. The error (obtained from the equation) discusses the difference between predicted and target values [21,23].

(3) $$E=\frac{1}{2}\sum {\left(X_{j}-Y_{j}\right)}^{2}$$

On behalf of this error signal, the next phase of MBNN is started, where the error signal is used for back calculation to adjust the values of weights and biases that were initially applied randomly; this is achieved by taking the derivative of the activation function, and then the architecture of the network is changed; for instance, the number of neurons in each layer or the number of layers is changed, and the number of cycles is also increased. Then, the feedforward process is repeated until errors are minimised and the network is optimised for precise results [21]. This process is carried out through the “Gradient Descent” method. The change in weight values depends on the error function “E” and the learning rate “η” applied [16]. This correction is calculated from Equation (4).

(4) $$\Delta W_{ji}=-\eta \frac{\delta E}{\delta W_{ji}}$$

As seen from Equation (4), if a higher learning rate value is applied, then abrupt changes in the value of weights would come out during each iteration if higher values of weights are used initially; this would lead to a prolonged process to achieve an optimised neural network model. On the other hand, with a small value of learning rate and small values of weights initially applied, training cycles are increased with prolonged training time, but they can proceed to a more optimised neural network model.

### 2.2. Transfer Function

The activation function (shown in Table 1) in an ANN model acts as a link between successive layers, processing the summation results and forwarding them to the next layer. The choice of activation function depends on the problem at hand and the normalisation of input parameters. Different activation functions are applied between various layers of the ANN model. The “log-sigmoid” activation function is used between the input layer and the hidden layer when the input data are normalised between 0 and 1. For the last two layers (hidden layer and output layer), the “linear” activation function is used when the normalisation process yields results between −1 and 1. In prediction or pattern identification cases, where the normalisation result is based on decision making, the “hyperbolic tangent” function can be used between all layers. In such cases, the “Gaussian” activation function is employed for the output layer, while the “hyperbolic tangent” activation function is used for the hidden layer. While no specific rule exists for selecting activation functions and data processing in training ANN models, certain commonly used parts have advantages and limitations. The sigmoid function provides non-linearity and an output range of 0 to 1, but it faces vanishing gradients and output saturation issues. The hyperbolic tangent (Tanh) function introduces non-linearity, it has a zero-centred output, and it offers stronger gradients, but it is also susceptible to vanishing gradients and lacks finite bounds. The Rectified Linear Unit (ReLU) is widespread in deep learning due to its sparsity, efficiency, and avoidance of vanishing gradients. Still, it can lead to dead neurons, and it lacks an upper bound. The softmax function is commonly used in the output layer for multi-class classification tasks, thus providing class probabilities. Still, it can be sensitive to large input values, and it assumes class independence. It is essential to carefully consider the advantages and limitations of each activation function when selecting them based on the task and network architecture to ensure optimal performance and stability in the ANN model.

### 2.3. MBNN Model Creation

For the prediction of flexural strength, shear strength, and the first crack load of the beam, the Multilayer Backpropagation Neural Network was selected. Previous studies found that the neural network’s performance is based on a sufficient sample database; the number of hidden layers, the number of neurons in both the input and hidden layers, and the initial values of weights and bias are assigned. Moreover, the activation function, error function, and learning program were chosen to define the network learning rate and its performance. Therefore, from the literature review, the suggestion given for the most optimised neural network for the best performance and a fast learning rate include the following points [20,24]:

1. Initial values for weights and biases should be assigned between −0.5 and 0.5.
2. In the hidden layer, the number of neurons should be double the amount of neurons in the input layer.
3. The activation function for the first two layers (input layer and hidden layer) should use the sigmoid activation function, while the output layer hyperbolic tangent activation function should be used.

Determining the ideal number of hidden layers and neurons in an Artificial Neural Network can significantly impact its predictive capabilities. Still, it is a challenging task requiring experimentation and fine-tuning. Increasing the number of layers or neurons can enhance the network’s ability to learn complex patterns but may lead to overfitting without proper regularisation. Conversely, reducing the number of layers or neurons simplifies the network but may result in underfitting. Cross-validation and grid search can be employed to find the optimal configuration. Cross-validation involves dividing the dataset into subsets, training and evaluating the network multiple times, and comparing performance across different configurations. Grid search systematically explores various hyperparameter combinations and selects the best configuration based on the validation set performance. Both methods facilitate a balanced architecture that maximises prediction accuracy on unseen data. However, it is important to note that the optimal architecture depends on the specific dataset and problem, thus necessitating careful evaluation and experimentation.

### 2.4. Sample Database 

**Figure 3:**
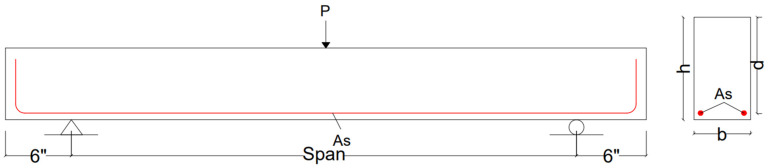
Beam cross-section details.

In total, 110 sample databases were included in this network training, testing, and validation process. Data were based on a simply supported rectangular beam without shear reinforcement (as shown in Figure 3), for which the sampling was performed in the UET Taxila concrete laboratory under the supervision of Prof. Dr. Ayub Elahi [25]. The ranges of the database are listed in the Table 2 below. The experimental results calculation procedure is explained in Appendix A. 

### 2.5. Data Processing

ANN performance is based on the database quality (input and output values). To reach optimisation, the network database should be normalised to make the network’s training process more efficient. After training, data can be denormalised for comparison with the target/output values, as the database has different units for both the input and output parameters. Hence, normalisation is performed to convert them into unitless values. Therefore, the network database needs to be normalised between two upper and lower values to avoid a low learning process. This process can be achieved using some built-in MATLAB functions in the Neural Network code, but if it is conducted before MATLAB data loading, it enables the user to control the model. In this model, data are normalised between 0.1 and 1 using Equation (5).

(5) $$\;x{}^{\prime }=\frac{\left(x-x_{\min}\right)}{\left(x_{\max}-x_{\min}\right)}\left(u-l\right)+l$$

where $\;x{}^{\prime }$ is the new, normalised value, x is the original value, u is the upper limit for normalisation, and l is the lower limit for normalisation.

### 2.6. Model Input and Output

In refining the model, input and output parameters (as displayed in Table 3) were meticulously chosen by systematically comparing results across diverse input variations. The selection of these parameters was contingent upon achieving optimal outcomes. Careful consideration was given to discern those specific input and output configurations that consistently yielded superior results, thus ensuring the model’s efficacy and reliability in generating optimal outcomes [26,27].

### 2.7. Division of Database

Data are divided into three subsets: (1) training, (2) validation, and (3) testing. The training dataset is used for the gradient update and weights and bias values, while the validation dataset calculates the error function during the training process and backpropagation. The testing set monitors different network models during the training process. Moreover, test subsets are used to calculate the difference between errors calculated by the testing subset after performing a number of iterations and a validation process to review the division of the database. If there is a difference, then the database division is varied based on trial and error to minimise that difference. Moreover, in our model, data are divided into 70% for training, 15% for validation, and 15% for testing. For division out of four, the MATLAB command “dividerand” is used, which divides the database randomly instead of user control, because this leads to a more efficient and optimised model [28,29]. 

### 2.8. The Functionality of the ANN Model

The ANN model (with functional parameters mentioned in Table 4) is trained as a multi-layer model that is coded in MATLAB using the Levenberg–Marquardt algorithm with a free forward backpropagation method [4,19,20]. The key aspects of training are summarised as follows:

(1) The training process involves dividing the database into three subsets using a random method. In total, 70% of the data is used for training, 15% is used for validation, and the remaining 15% is used for testing.
(2) In total, 1000 epochs/cycles are selected to train the ANN model, and the training is stopped if either of the following conditions is met: (a) a maximum of 100 validation failures occur, or (b) the minimum performance learning slope becomes 10^−8^.
(3) The error value of the correlation factor (R), the mean absolute error (MAE), and the mean squared error (MSE) are used to select the optimised ANN model [21,22,23,24]. These measures are expressed analytically by Equations (6)–(8), respectively, as referenced from the literature.

(6) $$R=\frac{\sum_{i=1}^{n}\left(X_{i}-\bar{X}\right)\left(Y_{i}-\bar{Y}\right)}{\sum_{i=1}^{n}{\left(X_{i}-\bar{X}\right)}^{2}\sum_{i=1}^{n}{\left(Y_{i}-\bar{Y}\right)}^{2}}$$

(7) $$\mathrm{MSE}=\frac{\sum_{i=1}^{n}\left(X_{i}{}^{2}-Y_{i}{}^{2}\right)}{n}$$

(8) $$\mathrm{MAE}=\frac{\sum_{i=1}^{n}\left(X_{i}-Y_{i}\right)}{n}$$

where $\bar{X}=\frac{\sum_{1}^{n}\left(X_{i}\right)}{n}$ and $\bar{Y}=\frac{\sum_{1}^{n}\left(Y_{i}\right)}{n}$, which are averages of the measured ($Y_{i}$) and predicted ($X_{i}$) outputs, while n is the number of the sample data in the database. To obtain an optimised ANN model, the value of “R” should be highest (approaching 1), while the values of “MSE” and “MAE” should be lowest [30].

To overcome the problem of overfitting, the gradient descent methodology is used to converge values of weights and biases, while, at the same time, early stopping criteria, as defined in the functionality of ANN, is employed to avoid overfitting [31,32,33].

## 3. Research Methodology

**Figure 4:**
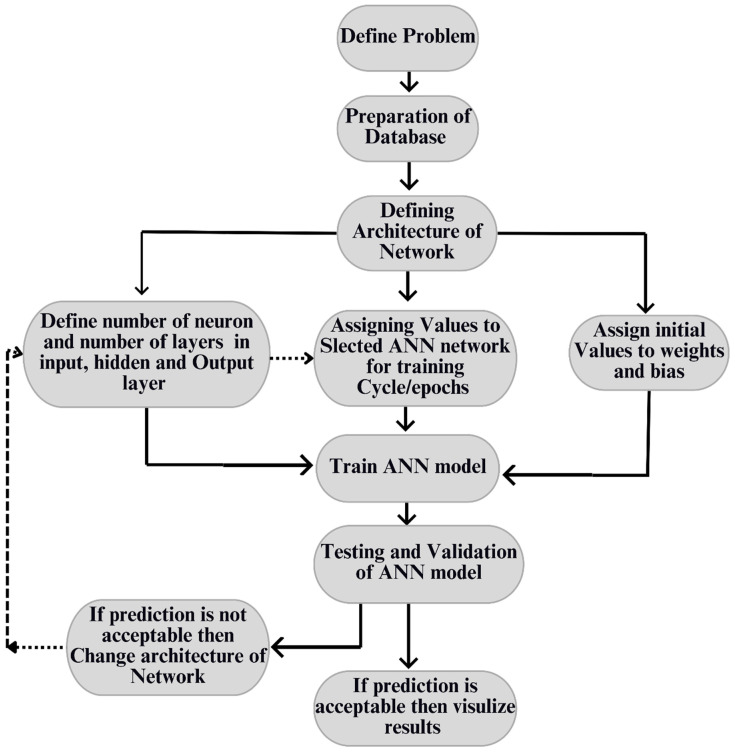
Research methodology layout.

Concrete waste is produced due to research work, and it participates in global warming to some extent as concrete is also a source of temperature increase. To overcome this, on behalf of previous sample data, one of the soft computing techniques, an Artificial Neural Network, is used to obtain optimised sample data results to reduce the number of physical concrete beam sample creations. For this research, the methodology adopted is displayed in the flow chart in Figure 4 to develop a green and economical solution. 

## 4. Results and Discussion

ANN model results are compared based on the regression curve and the coefficient of determination. Based on these values, regression values are in the range of 0.92 to 0.97 for all of the sample data against the first crack, flexural strength, and shear strength, while the coefficient of determination is above 0.94, which is an acceptable range. While running the training of the network, every time, results predicted by the model would be different, because each time, the weights and biases adjusted from the learning and error function are different from the previous trained model results. Therefore, to obtain optimised results, train the model at least three times and then obtain predicted results against your test input parameters. 

**Figure 5:**
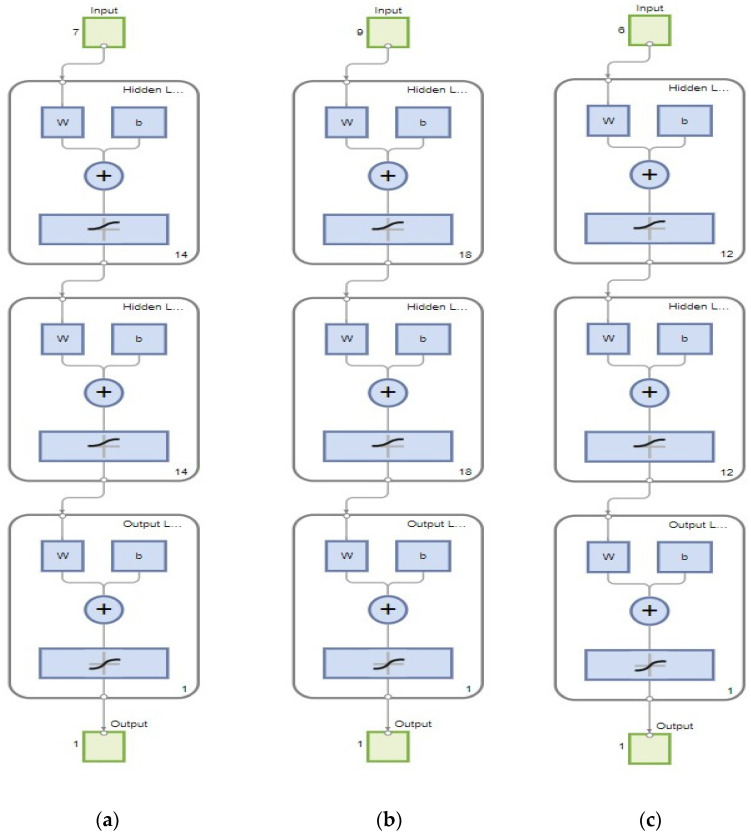
(**a**) Artificial Neural Network diagram for first crack load, (**b**) Artificial Neural Network diagram for flexural strength, (**c**) Artificial Neural Network diagram for shear strength.

The network diagram of the ANN model, as listed in Figure 5, shows the number of neurons in the hidden layer and output layer, along with the input data variables and output expected. 

**Figure 6:**
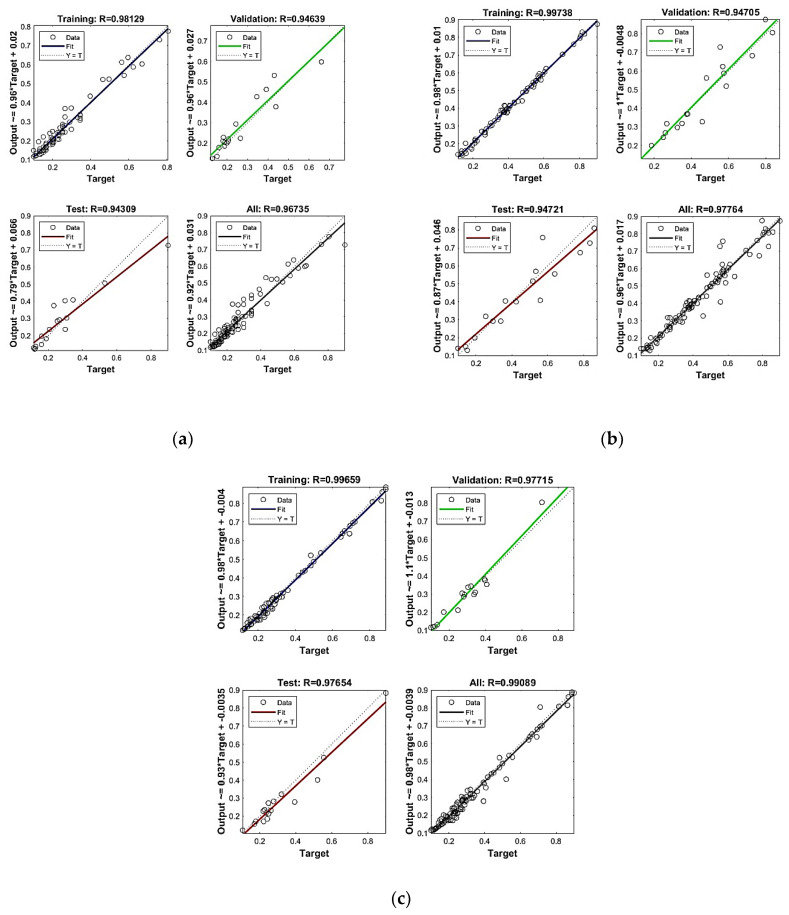
(**a**) ANN model results for first crack load prediction, (**b**) ANN model results for flexural strength prediction, (**c**) ANN model results for shear strength.

Regression analysis results (as shown in Table 5) for the first crack load, flexural strength, and shear strength model are listed in Figure 6; they show optimised regression values, like for the first crack load, which gives R = 0.981 for training. In contrast, for all (training, testing, and validation combined), R = 0.967, which is close to 1 and means that the predicted results are more concise. Similarly, flexural strength gives R = 0.997 for training. In contrast, for all (training, testing, and validation combined), R = 0.977 is close to 1, which is also within range and a more precise prediction, and for shear strength, the model gives R = 0.997 for training. In contrast, for all (training, testing, and validation combined), R = 0.991 is close to 1, which is also within range and a more precise prediction.

**Figure 7:**
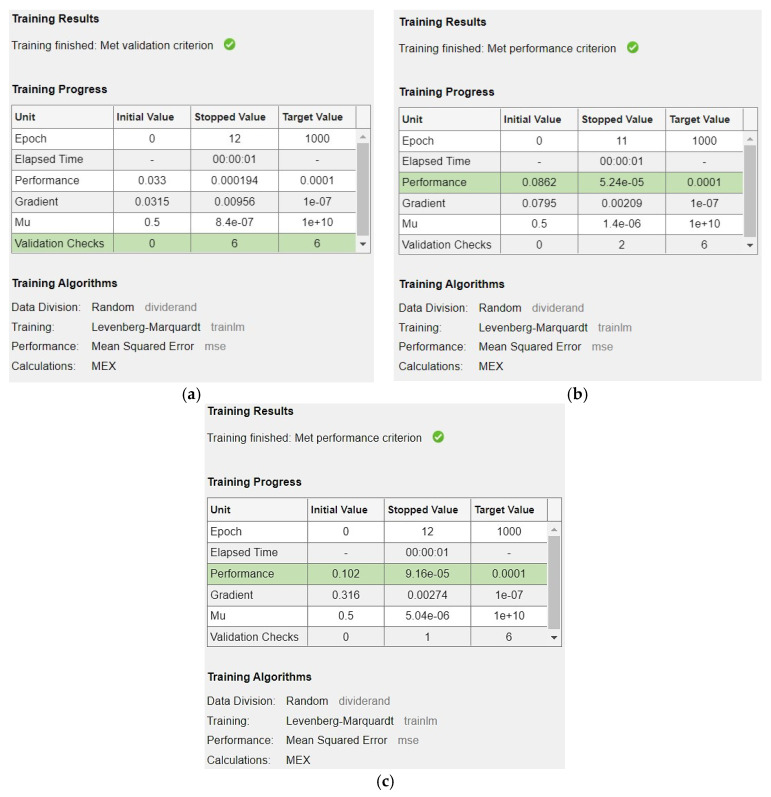
(**a**) Training results for the first crack load model, (**b**) training results for the flexural strength model, (**c**) training results for the shear strength model.

Similarly, the training results of the network are displayed in Figure 7, which shows which criteria have been met first and stopped the training process. It also shows how many iterations/epochs were completed while training the network. All of these stopping criteria are mentioned in the model creation procedure. 

### 4.1. Behaviour of First Crack Load Based on a/d

**Figure 8:**
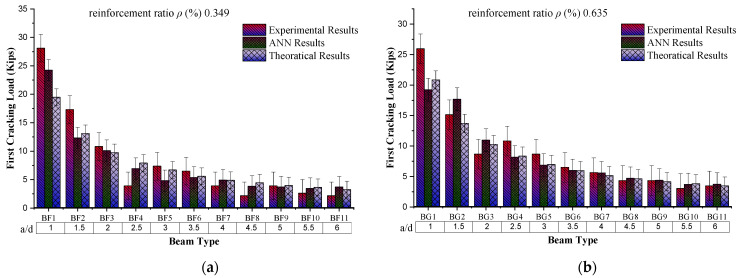

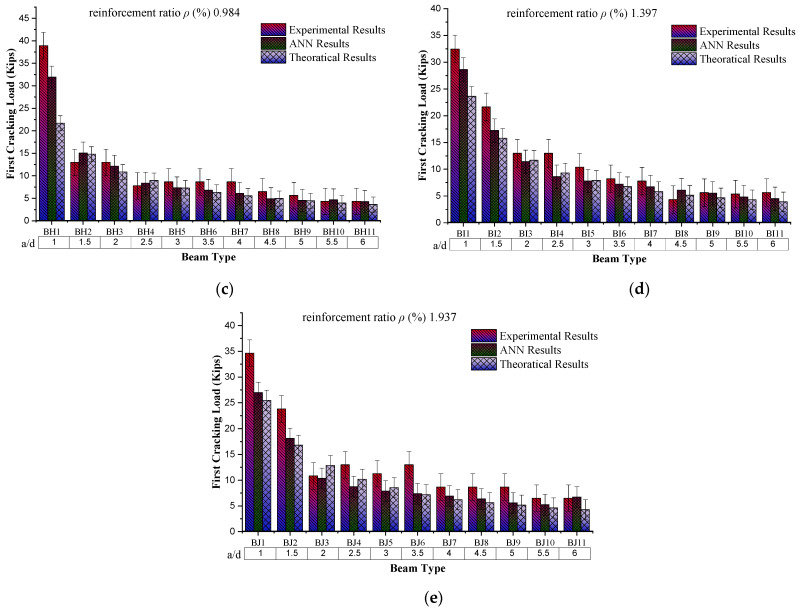
Comparison of fist cracking load with respect to shear span to effective depth ratio (a/d). (**a**) Group BF, comparison of first cracking load results with shear span to effective depth ratio (a/d) by keeping reinforcement ratio constant to 0.349. (**b**) Group BG, comparison of first cracking load results with shear span to effective depth ratio (a/d) by keeping reinforcement ratio constant to 0.635. (**c**) Group BH, comparison of first cracking load results with shear span to effective depth ratio (a/d) by keeping reinforcement ratio constant to 0.984. (**d**) Group BI, comparison of first cracking load results with shear span to effective depth ratio (a/d) by keeping reinforcement ratio constant to 1.397. (**e**) Group BJ, comparison of first cracking load results with shear span to effective depth ratio (a/d) by keeping reinforcement ratio constant to 1.937.

The behaviour of the first cracking load with the increasing shear span to an adequate depth (a/d) ratio while keeping the reinforcement ratio constant to 0.349% shows a decreasing trend. From the experimental results (from graph Figure 8), for the “a/d = 1”, the first cracking load is 28.1 kip, while for “a/d = 6”, this load value is reduced to 2.16 kip. The possible reason for this decreasing trend is the increase in the span length of the beam with increasing shear span to an effective depth ratio because of the constant value of “d”. Through comparison of the experimental results, ANN and theoretical results also show a decreasing trend for the same sample. ANN results from the comparison with experimental results give the coefficient of determination as 0.9404, which offers a more precise model for prediction against the given input parameters.

### 4.2. Behaviour of First Crack Load Based on the Reinforcement Ratio

**Figure 9:**
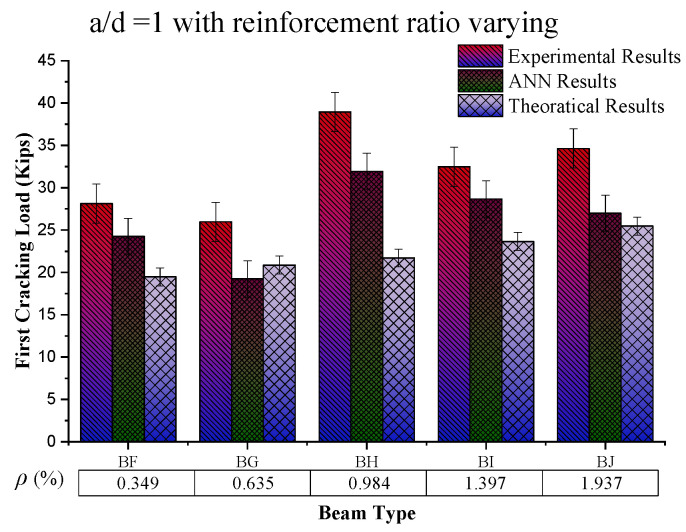
Comparison of first cracking load results by increasing reinforcement ratio (%) by keeping shear span to effective depth ratio (a/d) constant.

The constant value of the shear span to the effective depth ratio (a/d = 1) with an increasing reinforcement ratio (%) (from the graph in Figure 9) shows an increasing trend at up to 0.984% reinforcement ratio, which then decreases among the beam samples, while for the same sample value of experimental results, ANN results and theoretical results go on a decreasing trend, respectively. The possible reason for this increasing behaviour of first cracking load with increasing *ρ* (%) is the steel strength and the bond of concrete with steel, because during this condition, steel yields first and then concrete, which results in increased the load-carrying capacity of the beam. On the other hand, a further increase in the reinforcement ratio caused by reinforced beams results in brittle failure, causing a decrease in the beam’s load-carrying capacity. 

### 4.3. Behaviour of Flexural Strength Based on a/d

**Figure 10:**
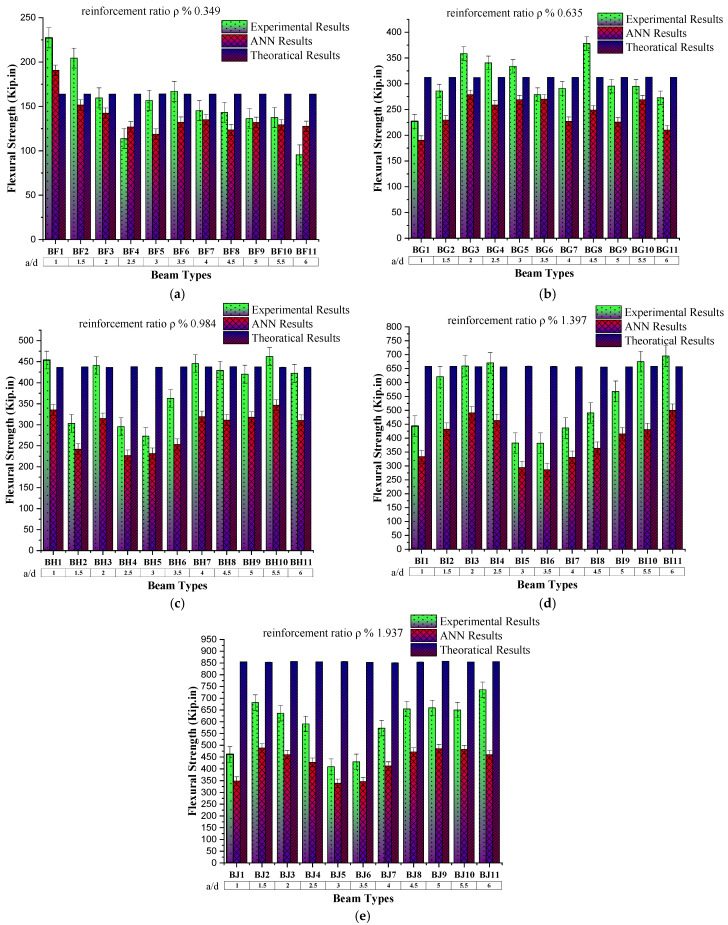
Comparison of flexural strength with respect to shear span to effective depth ratio (a/d). (**a**) Group BF, comparison of flexural strength results with shear span to effective depth ratio (a/d) by keeping reinforcement ratio constant to 0.349. (**b**) Group BG, comparison of flexural strength results with shear span to effective depth ratio (a/d) by keeping reinforcement ratio constant to 0.635. (**c**) Group BH, comparison of flexural strength results with shear span to effective depth ratio (a/d) by keeping reinforcement ratio constant to 0.984. (**d**) Group BI, comparison of flexural strength results with shear span to effective depth ratio (a/d) by keeping reinforcement ratio constant to 1.397. (**e**) Group BJ, comparison of flexural strength results with shear span to effective depth ratio (a/d) by keeping reinforcement ratio constant to 1.937.

The flexural strength of the reinforced concrete beam with a constant steel reinforcement ratio (ρ) 0.349% and varying the shear span to an effective depth ratio (a/d) shows a decreasing trend (as scene in Figure 10) with an increase in a/d. The same trend was observed in the case of ANN-predicted results. The prediction accuracy of ANN results is checked by finding the coefficient of determination by comparing the predicted results against specific input data with experimental results of that input data physical model, which is 0.9756.

### 4.4. Behaviour of Flexural Strength Based on Reinforcement Ratio

**Figure 11:**
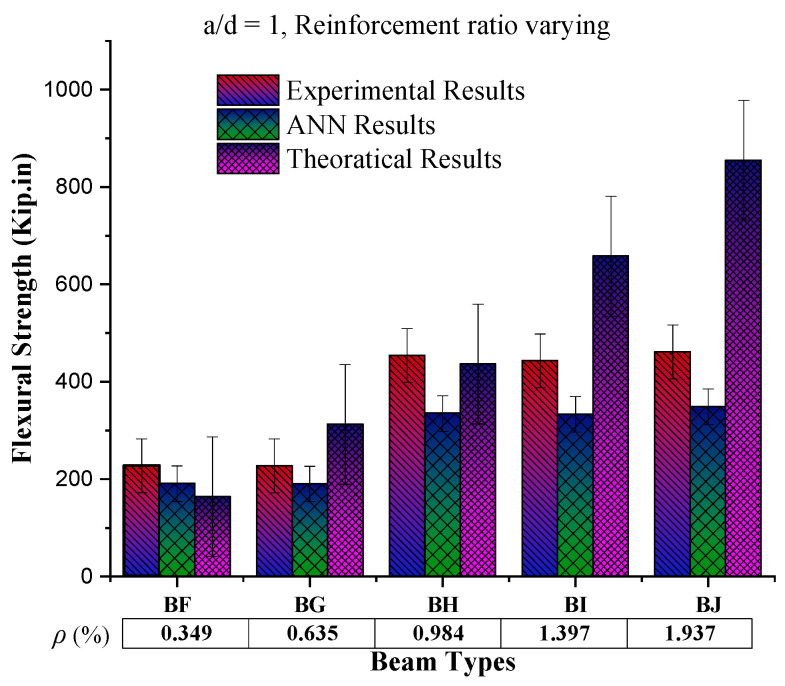
Comparison of flexural strength results by increasing reinforcement ratio (%) by keeping shear span to effective depth ratio (a/d) constant.

To check the effect of reinforcement on the beam by keeping the shear span effective depth ratio (a/d) constant, the experimental results (as scene in Figure 11) show an increase in flexural strength up to the reinforcement ratio 0.984%, which later shows the marginal change in flexural strength. At the same time, the ANN-predicted results against these input parameters show the same trend with increasing reinforcement. Furthermore, ANN-predicted results are close to the experimental results, which enables the applicability of the ANN model for the flexural strength or ultimate load prediction of the reinforced concrete member.

### 4.5. Behaviour of Shear Strength Based on a/d

**Figure 12:**
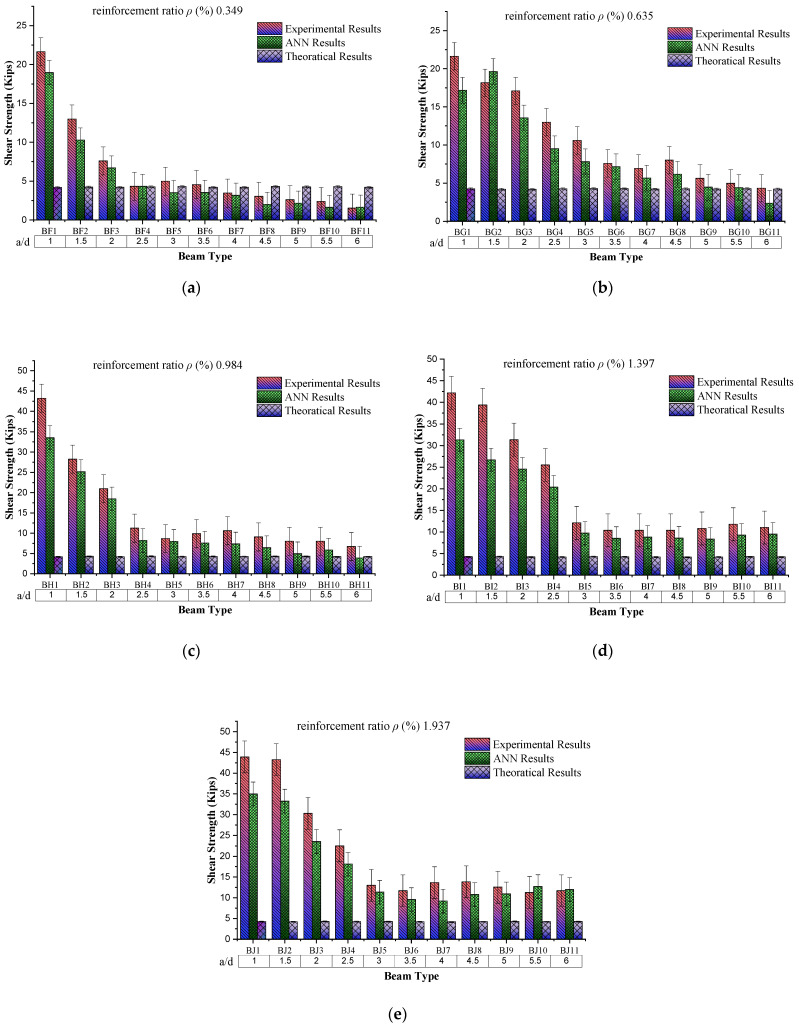
Comparison of shear strength results with respect to shear span to effective depth ratio (a/d). (**a**) Group BF, comparison of shear strength results with shear span to effective depth ratio (a/d) by keeping reinforcement ratio constant to 0.349. (**b**) Group BG, comparison of shear strength results with shear span to effective depth ratio (a/d) by keeping reinforcement ratio constant to 0.635. (**c**) Group BH, comparison of shear strength results with shear span to effective depth ratio (a/d) by keeping reinforcement ratio constant to 0.984. (**d**) Group BI, comparison of shear strength results with shear span to effective depth ratio (a/d) by keeping reinforcement ratio constant to 1.397. (**e**) Group BJ, comparison of shear strength results with shear span to effective depth ratio (a/d) by keeping reinforcement ratio constant to 1.937.

Beam sample group division is based on the reinforcement ratio, which varies from 0.349 to 1.937. In contrast, the shear span/depth ratio increases from 1 to 6 with an increment of 0.5 in each sample group. The behaviour of shear strength with the increasing shear span to adequate depth (a/d) ratio while keeping the reinforcement ratio constant to 0.349% shows a decreasing trend (as shown in Figure 12). From the experimental results for the “a/d = 1”, the shear strength is 21.64 kip, while for “a/d = 6”, this shear value is reduced to 1.52 kip. The possible reason for this decreasing trend is the increase in the span length of the beam with increasing shear span/depth ratio because of the constant value of “d”. When comparing the experimental results, the ANN and theoretical results also show a decreasing trend for the same sample. The decrease in experimental results from theoretical results may be due to an increase in the beam’s span length, resulting in the reduction of load-carrying capacity. The same trend is observed in all sample groups, as shown in the graphs.

### 4.6. Behaviour of Flexural Strength Based on Reinforcement Ratio

**Figure 13:**
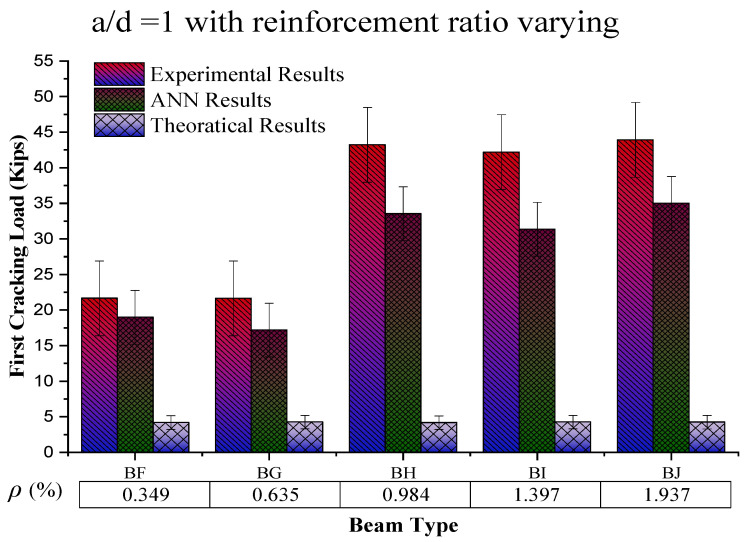
Comparison of shear strength results by changing ρ (%) and “a/d” constant.

The constant value of shear span/depth ratio (a/d = 1) with increasing reinforcement ratio (%) shows an increasing trend up to 0.984% reinforcement ratio (as scene in Figure 13). Then, it decreases among the beam samples, while for the same sample value of experimental results, the ANN results and theoretical results go on a decreasing trend, respectively. The possible reason for this increasing behaviour of shear strength with increasing ρ (%) is steel strength and the bond of concrete with steel because during this condition, steel yields first and then concrete, which results in increased load-carrying capacity of the beam. On the other hand, a further increase in the reinforcement ratio caused by reinforced beams results in brittle failure, causing a decrease in beam load-carrying capacity.

## 5. Conclusions

This study explains the development of a knowledge-based structural analysis model capable of predicting RC structural responses. The ANN model was developed based on the Multilayer Backpropagation Neural Network methodology, which enables the prediction of the behaviour of nonlinear elements. Input parameters are selected based on physical model preparation, and the predicted results are compared with the experimental results of the physical model. The predicted results are close to the experimental results.

1. The linear correlation coefficient “R” for the first cracking load stands at 0.96735. In contrast, the coefficient of determination “R^2^” for this parameter amounts to 0.9404—both values nearing 1—indicating highly accurate predictions closely resembling experimental findings.
2. In terms of flexural strength, the linear correlation coefficient “R” registers at 0.97764, and the coefficient of determination “R^2^” is at 0.9756. These values, which are in proximity to 1, affirm the precision of the predictions, closely mirroring experimental data.
3. As for shear strength, the linear correlation coefficient “R” is 0.99089, and the coefficient of determination “R^2^” is 0.9787. These figures, which are close to 1, signify the model’s ability to provide highly precise predictions, closely aligning with experimental outcomes.

In contrast to conventional methodologies for predicting the structural response of reinforced concrete components, soft computing techniques—specifically, the ANN model—stand out by accurately projecting the behaviour of RC structural components, regardless of their geometric complexity or various loading conditions. Once the ANN model is adequately trained using a relevant sample database, it demonstrates a remarkable ability to forecast the behaviour of RC structural components at the ultimate limit state (ULS) independent of the material properties and mechanics underlying the member. Furthermore, the ANN model can seamlessly integrate with professional design software for nonlinear analysis, enabling precise predictions of structural member responses at the ultimate limit state under both simple and intricate load scenarios. This integration significantly reduces analysis time and enhances the accuracy of predictions compared to the traditional approaches dictated by current design codes.

### Future Work Direction

The ANN model can be used with professional design software for nonlinear analysis to predict the response of structural members at the ultimate limit state under simple or complex loading without requiring analysis time, and it can predict accurate results compared to conventional current design codes. Moreover, new research can proceed by varying database conditions and input parameters.

## Figures and Tables

**Table 1:** Transfer function types [21].

Sr. No.	Transfer Function	Formula	Range	Graph
1	Identity (linear)	f(x) = x	(−∞, +∞)	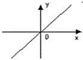
2	Step (threshold)	f(x) = 0 if 0 > x f(x) = 1 if x ≥ 0	[0, +1]	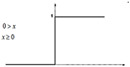
3	Piecewise Linear	f(x) = 0 if x ≤ x_min_ f(x) = mx + b if x_max_ > x > x_min_ f(x) = 1 if x ≥ x_max_	[0, +1]	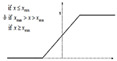
4	Sigmoid (Logistic)	$f\left(x\right)=\frac{1}{1-e^{-x}}$	(0, +1)	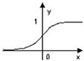
5	Hyperbolic	$f\left(x\right)=\frac{e^{x}-e^{-x}}{e^{x}+e^{-x}}$	(−1, +1)	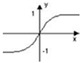
6	Gaussian	$f\left(x\right)=\frac{1}{\sqrt{2\pi \sigma }}e^{a}$ $a=\frac{-{\left(x-\mu \right)}^{2}}{2\sigma^{2}}$	(0, +1)	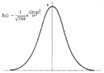

**Table 2:** Range of sample database for network training.

	b	d	Av/d	ρ_1_	a_1_	fy	fc	L	M_f_	P_cr_
Units	inch	inch		%		psi	psi	inch	lb.in	lb.
Max	7	12.5	6	2.057	2.91	77,222	8339	126	1,047,084	54,574
Min	6	10.5	1	0.349	0.37	70,727	7697	21	95,400	2160
SD	0.502	1.004	1.588	0.582	0.768	2599.74	157.47	33.35	237,180.73	10,268.58
Avg.	6.5	11.5	3.5	1.099	1.389	74286	8024.29	85.5	483,318.08	12,606.42
COV	0.077	0.087	0.454	0.529	0.553	0.035	0.0196	0.39	0.49	0.815

**Table 3:** Model input and output parameters.

Model Name	Input Parameters	Output Parameter
FCL	b, d, av/d, h, SL, fy, fc	First Cracking Load
FS	b, d, av/d, ρ, SL, a1, Pu, fy, fc	Flexural Strength
SS	b, d, av/d, Pu, fy, fc	Shear Strength

**Table 4:** Functional parameters for Artificial Neural Network model creation.

Character	Symbol	Value	Description
Network Name	MBNN		Multilayer Backpropagation Neural Network.
Database	Input	7 and 9	
	Output	1	
Hidden Layers	H1	Double	As from the literature, the most efficient model uses double the number of neurons in the hidden layer compared to the input layer.
	H2		
Hidden Neurons	H1	14 and 18	
	H2	14 and 18	
Activation Function	Input Layer	Log-sigmoid	Although the six activation functions listed in the above table can be used from previous research, these are recommended for obtaining an efficient network.
	Hidden Layer	Log sigmoid	
	Output Layer	Tain sigmoid	
Training and Learning Algorithms	Trainlm, learngdm		Levenberg–Marquardt.
Error Function		MSE, MAE, R	
Division	Training	70%	Data are divided randomly using the command dividerand.
	Validation	15%	
	Testing	15%	

**Table 5:** Error checks for the first cracking load model, flexural strength model, and shear strength model.

Parameters	First Cracking Load ANN Model	Flexural Strength ANN Model	Shear Strength ANN Model
MSE	0.0019	0.0017441	0.0017
MAE	0.0315	0.0234	0.016
R	0.96735	0.97764	0.99089
R^2^ (Coefficient of determination)	0.9404	0.9756	0.9787

**Figure A1:**
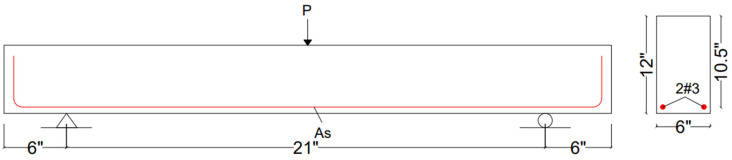
Loading pattern of RC beam for flexural behaviour.

**Figure A2:**
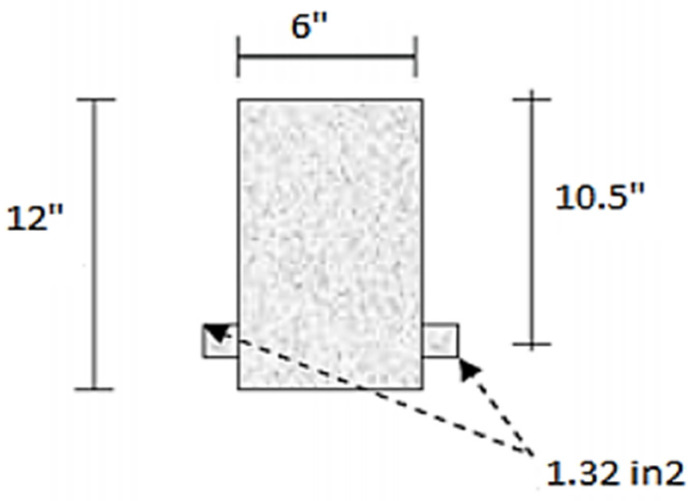
Concrete area replaced by reinforcement.

## Data Availability

Data will be provided on request.

## Abbreviations

b = width of beam
d = effective depth of beam
h = depth of beam
Av/d = shear span to effective depth ratio
ρ_1_ = steel reinforcement ratio
a1 = vitney block compressive area
fy = steel yield strength
fc = concrete compressive strength
L = length of beam
Mf = flexural strength of beam
Pcr = first cracking load
Vu = shear strength of beam
As = area of steel
SL = span length
FCL = model for first cracking load
FS = model for flexural strength
SS = model for shear strength

## Appendix A

Flexural behaviour of RC beam (in Figure A1) under applied loading of 43,280 lbs for the first sample BF1.

The moment developed due to the applied load is the maximum bending moment calculated by Equation (A1)

(A1) $$M=\frac{PL}{4}$$

$$M=\frac{43.280\times 21}{4}=227.22\;\mathrm{Kip}.\;\mathrm{in}$$

The gross moment of inertia of the beam can be calculated by Equation (A2)

(A2) $$I_{G}=\frac{bh^{3}}{12}$$

$$I_{G}=\frac{6\times 12^{3}}{12}=864\;in^{4}$$

The transformed moment of inertia of the concrete and steel was used to compute the stresses at the extreme tension fibre.

Transferred area of steel

(A3) $$n=\frac{E_{s}}{E_{c}}=6$$

(A4) $$A_{st}=n.\;A_{s}=6\times 0.22=1.32\;in^{2}$$

Distance from the top fibre to the neutral axis for the transferred inertial moment.

(A5) $$\bar{y}=\frac{A_{1}y_{1}+A_{2}y_{2}}{A_{1}+A_{2}}=\frac{1.32\times 10.5+72\times 6}{1.32+72}=6.081\;in$$

Distance of bottom fibre to transferred neutral axis

(A6) $$y_{b}=5.919\;in$$

Transferred moment of inertia

(A7) $$I_{tr}=\left(I_{G}+Ad^{2}\right)+A_{s}d^{2}=\left(864+72\times 0.081^{2}\right)+1.32\times 4.419^{2}=890.24\;in^{4}$$

Equating concrete tensile force to concrete rupture force

(A8) $$f_{ct}=f_{r}$$

(A9) $$\frac{My_{b}}{I_{tr}}=7.5\;\lambda \;\sqrt{f_{c}{}^{\prime }}$$

(A10) PL4ybItr=7.5 λ fc′

(A11) $$P_{cr}=\frac{4\times I_{tr}\times 7.5\;\lambda \;\sqrt{f_{c}{}^{\prime }}}{y_{b}L}$$

(A12) $$P_{cr}=\frac{4\times 890.24\times 7.5\times 1\times \sqrt{7840}}{5.919\times 21}=19.024\;Kip\;$$
